# Characterization of Ebola Virus Risk to Bedside Providers in an Intensive Care Environment

**DOI:** 10.3390/microorganisms9030498

**Published:** 2021-02-26

**Authors:** Mia J. Biondi, Lauren Garnett, Alexander Bello, Duane Funk, Philippe Guillaume Poliquin, Shane Jones, Kevin Tierney, Kaylie Tran, Robert A. Kozak, Anders Leung, Allen Grolla, Cory Nakamura, Geoff Soule, Charlene Ranadheera, Mable Hagan, Amrinder Dhaliwal, Darwyn Kobasa, Darryl Falzarano, Hugues Fausther Bovendo, Heinz Feldmann, Murray Kesselman, Gregory Hansen, Jason Gren, Todd Mortimer, Trina Racine, Yvon Deschambault, Jocelyn Edmonds, Sam Aminian, Ray Saurette, Mark Allan, Lauren Rondeau, John Huynh, Sharron Hadder, Christy Press, Christine DeGraff, Stephanie Kucas, Julie Kubay, Kim Azanarsky, Bradley W. M. Cook, BJ Hancock, Anand Kumar, Reeni Soni, Daryl Schantz, Jarrid McKitrick, Bryce Warner, Bryan D. Griffin, Xiangguo Qiu, Gary P. Kobinger, Dave Safronetz, Heidi Wood, Derek R. Stein, Todd Cutts, Brad Pickering, James Kenny, Steven Theriault, Liam Menec, Robert Vendramelli, Sean Higgins, Logan Banadyga, Guodong Liu, Md Niaz Rahim, Samantha Kasloff, Angela Sloan, Shihua He, Nikesh Tailor, Alixandra Albietz, Gary Wong, Michael Gray, Friederike Feldmann, Andrea Marzi, George Risi, James E. Strong

**Affiliations:** 1 Toronto Centre for Liver Disease, University Health Network, Toronto, ON M5G 2C4, Canada; mia.biondi@mail.mcgill.ca; 2 National Microbiology Laboratory, Public Health Agency of Canada, Winnipeg, MB R3E 3M4, Canada; lauren.garnett@canada.ca (L.G.); alexander.bello@canada.ca (A.B.); funk@cc.umanitoba.ca (D.F.); guillaume.poliquin@canada.ca (P.G.P.); shane.jones@canada.ca (S.J.); kevin.tierney@canada.ca (K.T.); kaylie.doan@canada.ca (K.T.); anders.leung@canada.ca (A.L.); allengrolla@gmail.com (A.G.); geoff.soule@canada.ca (G.S.); charlene.ranadheera@canada.ca (C.R.); mable.hagan@canada.ca (M.H.); darwyn.kobasa@canada.ca (D.K.); jason.gren@canada.ca (J.G.); yvon.deschambault@canada.ca (Y.D.); Christine.DeGraff@phac-aspc.gc.ca (C.D.); stephanie.kucas@canada.ca (S.K.); julie.kubay@canada.ca (J.K.); kimberly.azaransky@canada.ca (K.A.); bryce.warner@canada.ca (B.W.); bdjgriffin@gmail.com (B.D.G.); xiangguo.qiu@canada.ca (X.Q.); david.safronetz@canada.ca (D.S.); heidi.wood@canada.ca (H.W.); todd.cutts@canada.ca (T.C.); liammenec@riverviewcc.ca (L.M.); robert.vendramelli@canada.ca (R.V.); seanhiggins@shaw.ca (S.H.); logan.banadyga@canada.ca (L.B.); guodong.liu@canada.ca (G.L.); mdniaz.rahim@canada.ca (M.N.R.); samantha.kasloff@canada.ca (S.K.); angela.sloan@canada.ca (A.S.); shihua.he@canada.ca (S.H.); nikesh.tailor@canada.ca (N.T.); alix.albietz@canada.ca (A.A.); mcgray3@shaw.ca (M.G.); 3 Department of Medical Microbiology and Infectious Diseases, Faculty of Health Sciences, College of Medicine, University of Manitoba, Winnipeg, MB R3E 0J9, Canada; JMcKitrick@exchange.hsc.mb.ca (J.M.); bradley.pickering@canada.ca (B.P.); 4 Department of Anaesthesia, Faculty of Health Sciences, College of Medicine, University of Manitoba, Winnipeg, MB R3E 0Z2, Canada; 5 Department of Paediatrics & Child Health, Faculty of Health Sciences, College of Medicine, University of Manitoba, Winnipeg, MB R3A 1S1, Canada; Mkesselman@exchange.hsc.mb.ca (M.K.); TMortimer@exchange.hsc.mb.ca (T.M.); jedmonds@exchange.hsc.mb.ca (J.E.); saminian@exchange.hsc.mb.ca (S.A.); ray123@mts.net (R.S.); mark_allan@live.ca (M.A.); grantlaurenl@gmail.com (L.R.); j.huynh9@gmail.com (J.H.); shadder@exchange.hsc.mb.ca (S.H.); Cpress@exchange.hsc.mb.ca (C.P.); BJHancock@exchange.hsc.mb.ca (B.H.); akumar61@yahoo.com (A.K.); RSoni@exchange.hsc.mb.ca (R.S.); dschantz@exchange.hsc.mb.ca (D.S.); jkenny@exchange.hsc.mb.ca (J.K.); 6 Department of Laboratory Medicine & Pathobiology, University of Toronto, Toronto, ON M5S1A8, Canada; robert.kozak@mail.mcgill.ca; 7 National Centre for Foreign Animal Disease, Canadian Food Inspection Agency, Winnipeg, MB M3E 3M4, Canada; Cory.nakamura@inspection.gc.ca; 8 Medtronic Canada, Winnipeg, MB M3A 1R9, Canada; rimmidhaliwal@hotmail.com; 9 Vaccine and Infectious Disease Organization-International Vaccine Centre, University of Saskatchewan, Saskatoon, SK S7N 5E3, Canada; darryl.falzarano@usask.ca (D.F.); trinadracine@gmail.com (T.R.); 10 Centre de Recherche en Infectiologie, Centre Hospitalier Universitaire de Québec, Université Laval, Quebec City, QC G1V 4G2, Canada; hugues.fausther.bovendo@crchudequebec.ulaval.ca (H.F.B.); gary.kobinger@crchudequebec.ulaval.ca (G.P.K.); 11 Laboratory of Virology, Division of Intramural Research, National Institute of Allergy and Infectious Diseases, National Institutes of Health, Hamilton, MT 59840, USA; feldmannh@niaid.nih.gov (H.F.); feldmannfe@niaid.nih.gov (F.F.); marzia@niaid.nih.gov (A.M.); 12 Faculty of Critical Care, Royal University Hospital, Saskatoon, SK S7N 0W8, Canada; gregory.hansen@usask.ca; 13 Cytophage Technologies, Inc., Winnipeg, MB R3Y 1G4, Canada; Bradley.cook@canada.ca (B.W.M.C.); steven.theriault@canada.ca (S.T.); 14 Cadham Provincial Laboratory, Winnipeg, MB R3E 37J, Canada; Derek.Stein@gov.mb.ca; 15 Departement of Microbiology-Infectiology and Immunology, Université Laval, Québec City, QC G1V 0A6, Canada; garyckwong@hotmail.com; 16 Infectious Disease Specialist, P.C., Missoula, MT 59802, USA; Grisi@blackfoot.net

**Keywords:** Ebola, nosocomial infection, critical care, viral shedding, environmental contamination

## Abstract

Background: The 2014–2016 Ebola outbreak in West Africa recapitulated that nosocomial spread of Ebola virus could occur and that health care workers were at particular risk including notable cases in Europe and North America. These instances highlighted the need for centers to better prepare for potential Ebola virus cases; including understanding how the virus spreads and which interventions pose the greatest risk. Methods: We created a fully equipped intensive care unit (ICU), within a Biosafety Level 4 (BSL4) laboratory, and infected multiple sedated non-human primates (NHPs) with Ebola virus. While providing bedside care, we sampled blood, urine, and gastric residuals; as well as buccal, ocular, nasal, rectal, and skin swabs, to assess the risks associated with routine care. We also assessed the physical environment at end-point. Results: Although viral RNA was detectable in blood as early as three days post-infection, it was not detectable in the urine, gastric fluid, or swabs until late-stage disease. While droplet spread and fomite contamination were present on a few of the surfaces that were routinely touched while providing care in the ICU for the infected animal, these may have been abrogated through good routine hygiene practices. Conclusions: Overall this study has helped further our understanding of which procedures may pose the highest risk to healthcare providers and provides temporal evidence of this over the clinical course of disease.

## 1. Background 

The 2014–2016 epidemic of Ebola virus [1] in West Africa totaled 28,646 cases, with 11,323 deaths [2]. Since that time, there has been four additional smaller outbreaks in the Democratic Republic of the Congo [3]. Infection with the virus causes Ebola Virus Disease (EVD), with an overall case fatality rate of approximately 40% but outbreaks have shown lethality upwards of 90% [4,5,6]. While vaccines and therapies prove beneficial [7], like other acute viral infections, the best method for outbreak control is limiting transmission through patient isolation, quarantine measures, and infection control practices. During Ebola virus outbreaks the disease affects a disproportionate number of healthcare personnel. One report from the 2014–2016 outbreak predicted that healthcare providers were up to 32 times more likely to be infected than the general population, with approximately 50% of health worker infections made up of nurses and nursing aids [8]. 

In contrast to previous outbreaks, the 2014 epidemic resulted in 27 EVD patients being managed in 15 hospitals in nine resource-rich countries due to inadvertent importation of cases, or individuals being purposefully repatriated. Unfortunately, these imported cases also led to the first-ever nosocomial acquisitions in non-outbreak countries [4,9]. These transmission events emphasize the delicate balance between the need to provide care and the risk to the healthcare provider. The Centers for Disease Control (CDC) urges cautions regarding the risk associated with several procedures [10]; however, driven by medical necessity, many high-risk procedures have been performed in resource-rich settings [1,11]. 

Environmental sampling of healthcare facilities in outbreaks has been investigated and connected to possible transmission events [12], but there is a paucity of data examining environmental contamination during EVD in ICU care settings. Previous experimental studies have shown that Ebola virus (EBOV) persists on personal protective equipment (PPE) for at least 192 h and appears to show preference for stainless steel over plastic surfaces [13]. Additionally, work with EBOV-spiked whole blood or water has demonstrated that when left on surfaces to dry at temperatures mimicking climate-controlled healthcare settings, or conditions in West Africa; virus could be detected for up to 14 days [14]. In the work presented herein, we sequentially infected four non-human primates with EBOV in a fully equipped ICU within a Biosafety Level 4 (BSL4) in Winnipeg, Canada. The objectives of this study are two-fold: 1) To better understand which procedures during routine care posed the greatest risk to providers; and 2) to determine the amount of viral RNA accumulated on the surfaces at the end-point of the disease. 

## 2. Methods

### 2.1. Non-Human Primate Model of EBOV Infection 

Four Rhesus macaques (*Macaca mulatta*) were used as animal models of Ebola Virus Disease (EVD) [15]. Animal approval was granted by the National Microbiology Laboratory Animal Care Committee, under the Canadian Council of Animal Care*. The NHP was sedated using intramuscular and inhalation combination pharmacotherapy described by Polquin et al. [16]. Under ultrasound guidance pediatric surgeons and critical care physicians inserted two femoral arterial and venous central lines, at which point combination intravenous sedation was initiated. Nasal or oral intubation was initiated, with in-line suction. Ventilation was monitored at both the bedside or via on-site telemonitoring outside the BSL4. A nasogastric (NG) tube was inserted for feeding, and Foley urinary catheter inserted to allow for accurate output, and sampling of urine for analysis. After a period of stabilization, each NHP was infected with Ebola virus/H.sapiens wt/GIN/2014/Makona-C07 (EBOV, Genbank 128 accession number KT013257.3), by two divided intramuscular injections into the vastus lateralis of a planned dose of 1000 TCID50/mL. Bedside staff were equipped with BSL4 PPE from this point onwards, including positive pressurized suits with affixed umbilical air lines following BSL4 standard procedures. The NHP was sedated for the duration of the experiment, for animal comfort and the safety of staff.

### 2.2. Intensive Care Environment 

Each of the ICU experiments took place in a BSL4 laboratory with an average temperature of 21–22 °C, and a relative humidity of approximately 45%. The BSL4 laboratory’s ventilation system meets the requirements of the Canadian Biosafety Standard (2nd edition) [17] and Biosafety in Microbiological and Biomedical Laboratories (6th Edition) [18], including 100% non-recirculating fresh air supply, an air flow of 260 LPS, and −250 Pa differential pressure with respect to ambient. Directional airflow was from foot of bed toward head of bed and upwards to ceiling area to NHP’s right, head side. Two or more care providers in an isolated room mimicking an adult or pediatric ICU monitored the animal around-the-clock. These included nurses, critical care physicians, pediatricians, respiratory therapists, a pharmacist, ultrasound technicians, and BSL4-trained veterinary or scientific staff. Equipment for the ICU included a patient monitor (Intellivue MX800 Patient Monitor, Philips), ventilator (Avea Ventilator, CareFusion), pressure bags, a volumetric pump (Alaris SE Volumetric Pump), syringe pumps (Alaris CC Syringe Pump), an electric warming blanket (HotDog Veterinary Patient Warming System). Bedside diagnostic equipment for veterinary CBCs (VetScan HM5), blood gases (ABL 80 Flex Blood Gas Analyzer), biochemistry (Piccolo Blood Chemistry Analyzer), point-of-care glucometer (Aviva Accu-Chek), and bedside x-ray, ECG, and ultrasound were also available. All other disposable equipment were available at the bedside, i.e., IV solutions, lines, flushes, feed bags, suction equipment, nasal/oral/eye care supplies, sample collection devices and tubes, and medication, including emergency IV/IM sedation. A complete list of fluid replacement, medications/interventions, as well as details of ventilation, vital signs ranges, and fluid balance has previously been published [16]. Daily care for the animal included routine critical care assessments, interventions, and patient care (Table 1). 

### 2.3. Collection of Blood, Body Fluids and Clinical Swabs

Venous blood was collected prior to infection as a baseline for clinical monitoring. Following infection, whole blood was collected by needleless syringe through either the central line or arterial line and placed into plastic EDTA-microtubes (BD, Mississauga, ON, Canada), and stored at −80 °C for viral RNA analysis. Blood draws amounted to no more than 10% of the NHP circulatory volume over the course of the study, as per Canadian Council on Animal Care guidelines. Additionally, 1 mL of gastric residuals and urine were collected at least daily in two of four animals by gastric or needless syringe and stored in 2-mL cryovial (Sarstedt, St-Leonard, QC, Canada) at −80 °C for analysis. 

We also collected daily swabs from two of four animals (HydraFlock, Puritan Diagnostics, Guilford, ME, USA) by rubbing each designated site for 5–10 s and immediately immersing the swab and storing in 500 μL 1× phosphate-buffered saline, pH 7.4, with penicillin and streptomycin (PBS-P/S) and storing at −80 °C for analysis. These included: ocular, nasal, and buccal to assess risk when providing eye, nasal, or oral care, as the bedside staff did throughout the experiment. We also collected a rectal swab assessing risk for obtaining rectal temperature or suppository insertion, and a skin swab under the armpit to assess whether EBOV was present under the arm important for routine care or repositioning the animal. 

### 2.4. Environmental Sampling Post-Mortem 

The clinical duration of EVD was approximately seven days (mortality by 6.8 to 7.7 days). At the end of the experiment, dressed in full PPE, investigators swabbed bedside and equipment surfaces. HydraFlock (Puritan Diagnostics) or MedPro^®^ (Mississauga, ON, Canada). Cotton Tipped Applicator swabs were pre-moistened with PBS-P/S prior to swabbing a 30 cm by 30 cm sampling area for large surface areas (floor, bed area, screens, or countertops) or over the button areas of common touch points (syringe pumps, volumetric pump, switches on equipment) for 5–10 s, and immediately stored in PBS-P/S at −80 °C for later analysis. Investigators performed full-hand cleaning procedures in 5% MicroChem solution and/or 70% ethanol before and after swabbing. Surfaces swabbed were categorized into patient area and devices.

### 2.5. qRT-PCR

Viral RNA from blood, urine, gastric residuals, and animal swabs, as well as environmental swabs was extracted using the QIAamp viral RNA mini kit (Qiagen, Germantown, MD, USA) following manufacturer instructions. Virus inactivation and tube decontamination occurred within the BSL4 as described elsewhere [19]. The completion of viral extraction was done under Biosafety Level 2 conditions. One-step reverse transcription qPCR was carried out to measure cycle threshold (Ct) using two targets within the EBOV genome, namely the L-gene and the nucleoprotein (NP)-gene using Lightcycler 480 RNA Master Hydrolysis reagents (Roche Diagnostics) according to manufacturer’s instructions, with primers and probes, with a Ct of 36 or lower considered positive, and decreasing Cts correlate with increasing viral loads, all as previously described [19,20,21,22]. Inter-run variability was limited by using the same primers, probes and platform between runs and animals. 

## 3. Results

### 3.1. Ebola Virus Disease Outcomes in Non-Human Primates 

**Figure 1:**
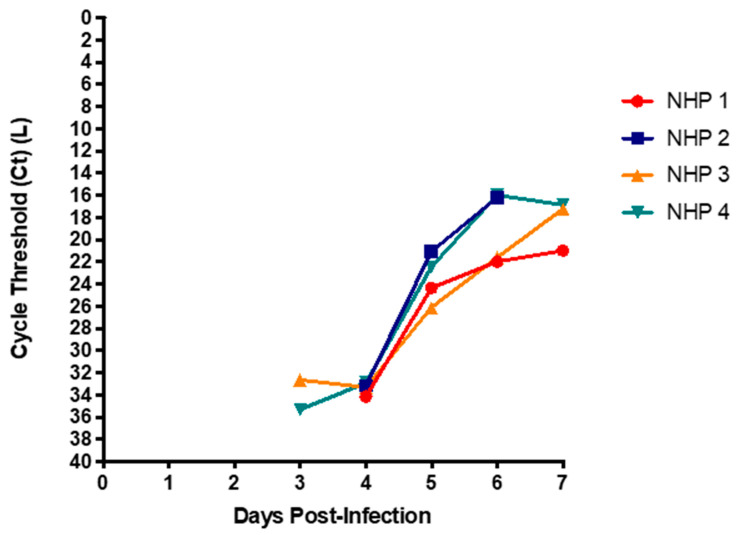
Cycle threshold (Ct) in blood during Ebola Virus Disease (EVD)**.** Blood was collected from four non-human primates 1–2 weeks before infection, and daily throughout the course of clinical disease. All samples were extracted, reverse transcribed, and then quantified using L-gene for EBOV RNA. Non-human primates (NHP) 1 (**●**), NHP 2 (■), NHP 3 (**▲**), and NHP 4 (**▼**).

The clinical course and outcome of all four animals in this experiment were remarkably similar despite the differences in viral challenge, treatment, and medical complications (previously published [16,23]). All Ebola-infected animals went through a period of initial compensated shock, occurring between 5 to 6 days post-infection (DPI) followed by uncompensated shock starting on 6 DPI, and leading to multi-system failure and death by 7 DPI, despite aggressive but judicious fluid, inotropic, and other intensive medical care. As shown in Figure 1, the pattern of Cts in each of the four animals in this series show remarkable similarities in onset, trajectory, as well as time to death.

### 3.2. Viral RNA Levels in Blood and Body Fluids 

Blood samples were collected daily via central venous or arterial line access following infection until the completion of the experiment. In all four studies viral RNA was detected in blood by 3 to 4 DPI, and Cts decreased until animal death (Figure 1). In order to evaluate the potential risk associated with completing routine fluid balance monitoring, assessments and labs, collecting urine samples for biochemical analysis, disposing of urine if a Foley catheter is inserted, or if replacing briefs; we monitored RNA in urine over the course of disease for the first two of four NHPs. We also hypothesized that measuring gastric residuals, assessing nasogastric (NG) placement by pH, or re-inserting an NG tube would also be a high-risk procedure, and therefore monitored RNA in gastric fluid over the course of disease. 

**Figure 2:**
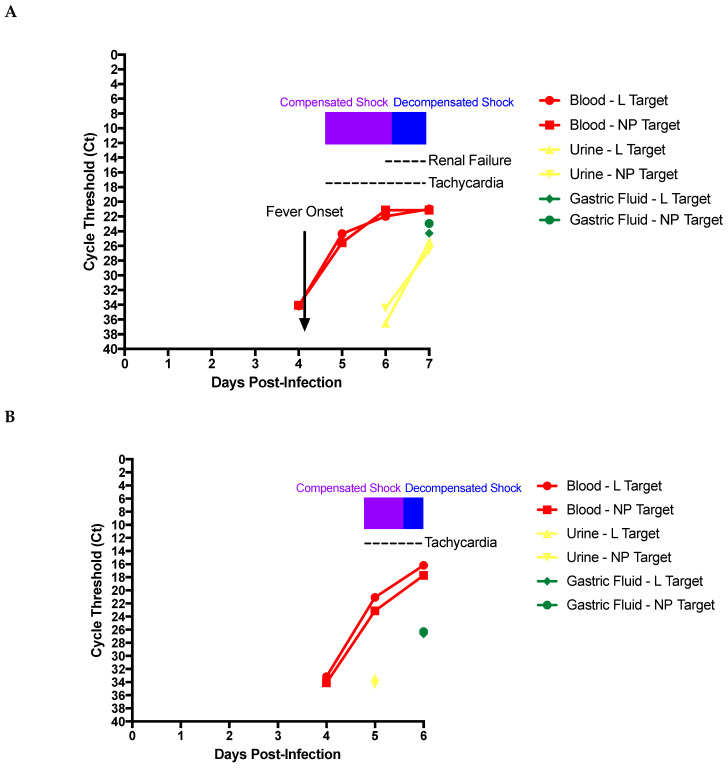
Detection of viral RNA in blood and body fluids during EVD. Blood, urine, and gastric residuals were collected 1–2 weeks before study initiation, and daily throughout the course of clinical disease. All samples were extracted, reverse transcribed, and then quantified using two targets for EBOV RNA quantification, blood L-gene (**●**) and NP- targets (■), urine L-gene (**▲**) and NP- targets (**▼**), and gastric residuals L-gene (♦), and NP-targets (**●**) for (**A**) NHP 1 and (**B**) NHP 2.

Viral RNA was detected in blood prior to other body fluids. In the first study, viral RNA was not detected in urine until 6DPI, with a mean Ct of 26 on 7DPI, as compared to 35.5 on 6DPI (Figure 2A). This change was in contrast to what was seen in blood where Cts were similar on 6DPI and 7DPI. However, EBOV from the syringed gastric fluid was detectable only on 7DPI. Similarly, viral RNA was detected in urine on 5DPI in the second animal. Unfortunately, we were unable to assess viral RNA in urine after this, as this animal became aneuric past this point in the disease process. As seen in the first study, detectable RNA was present in gastric fluid as of 6DPI in the second animal with a mean Ct of 26.5, in comparison to blood with a mean of 17 (Figure 2B). Urine for animals 3 and 4 was also positive at the time of death. 

### 3.3. Detection of EBOV RNA in Clinical Swabs 

**Figure 3:**
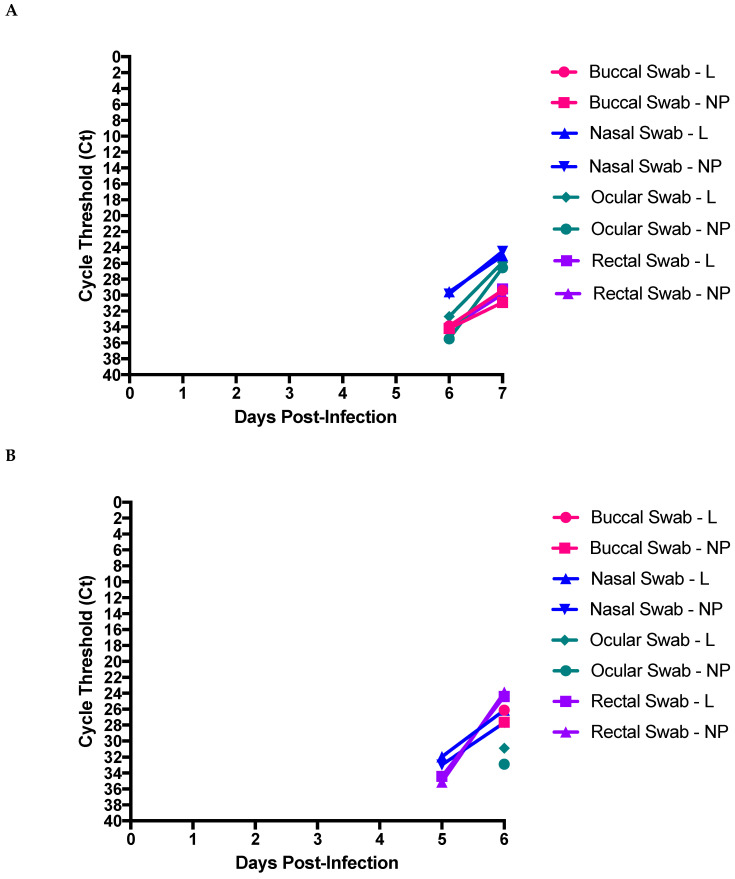
Detection of viral RNA in clinical swabs throughout disease course. Buccal, nasal, ocular, rectal, and skin swabs were collected 1–2 weeks before study initiation, and daily throughout the course of clinical disease. All samples were extracted, reverse transcribed, and then quantified using two targets for EBOV RNA, buccal L-gene (**●**) and NP- targets (■), nasal L-gene (**▲**) and NP- targets (**▼**), ocular L-gene (♦) and NP-targets (**●**), rectal L-gene (■) and NP-targets (**▲**), skin L-gene (**▼**), and NP-targets (♦) for (**A**) NHP 1 and (**B**) NHP 2.

We collected daily patient swabs to assess the potential risk of providing routine nursing care in an ICU setting. All swabs had detectable nucleic acid at 6DPI with the exception of the skin swab, however only the nasal swab had a Ct lower than 30 (Figure 3A). By 7DPI viral nucleic acid was detected in nasal, ocular, rectal, and buccal swabs, although each had significantly less than in blood. No viral RNA was detected on skin at 7DPI. Interestingly, in the second animal only the nasal and rectal swabs had detectable RNA at 5 DPI, while at 6DPI the rectal swab had the most amount of viral RNA (Figure 3B), followed the buccal/nasal swab, then ocular swab, with no detectable RNA in the skin swab 6DPI. Similar to the first study, significantly less viral RNA was present in the swabs as compared to the blood. 

### 3.4. Detection of EBOV RNA in Environmental Swabs 

**Figure 4:**
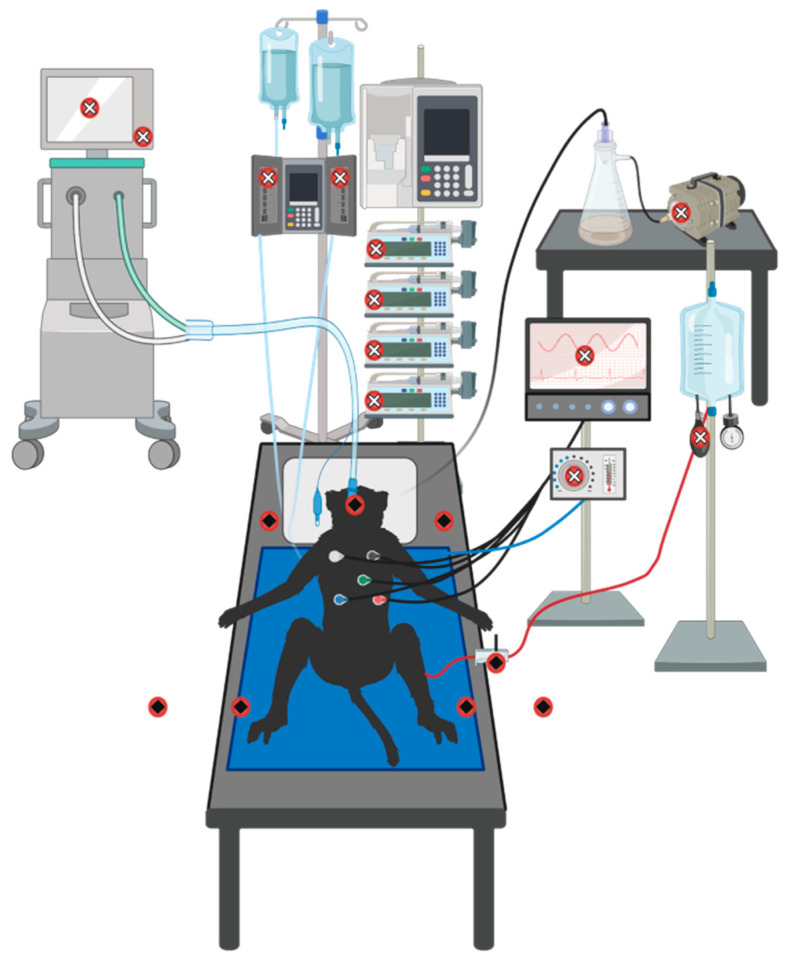
Environmental surface sampling. Swabbing occurred immediately post-mortem for both studies following NHP multi-system organ failure for 5–10 s per surface. The patient area (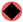) included the endotracheal tube (ETT), ART-line and CVP transducers, the right and left sides of the head of the bed, the right and left sides of the bed in line with the NHP, and the floor on either side of the bed. The device area (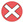) included the Avea Ventilator dial and entirety of screen, the Intellivue MX800 Patient Monitor screen, the Alaris SE Volumetric Pump, four Alaris CC Syringe Pumps, the pressure bulbs to maintain adequate pressure for ART-lines, the suction pump, and the HotDog Veterinary Patient Warming System on/off button (not shown).

**Figure 5:**
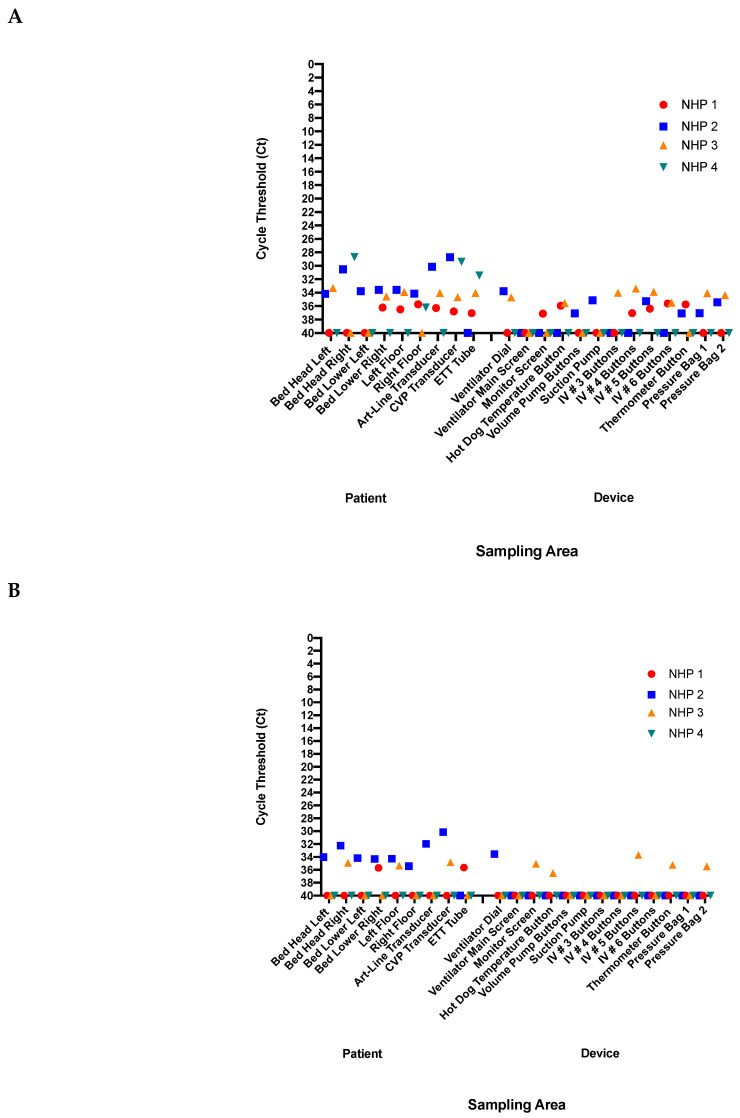
Detection of viral RNA in intensive care unit (ICU) environment at end-point. Following NHP deterioration, environmental surface swabs were collected by swabbing for 5–10 s per surface. Sampling was divided into the patient area including the endotracheal tube (ETT), ART-line and CVP transducers, the right and left sides of the head of the bed, the right and left sides of the bed in line with the NHP, and the floor on either side of the bed. The device area included the Avea Ventilator dial and entirety of screen, the Intellivue MX800 Patient Monitor screen, Alaris SE Volumetric Pump, four Alaris CC Syringe Pumps, the pressure bulbs to maintain adequate pressure for ART-lines, the suction pump, and the HotDog Veterinary Patient Warming System on/off button. All samples were quantified using qPCR for both (**A**) L-gene and (**B**) NP-targets in the patient and device area.

Viral nucleic acid was found on medical equipment and in the treatment environment during the recent West African Ebola epidemic [19,24]. Therefore, we sought to investigate which areas in our ICU had the highest viral burden. Areas were categorized as “patient” or “device” areas (Figure 4). Multiple surface areas had detectable viral nucleic acid in the immediate patient area (Figure 5A,B). Specifically, the head of the bed, the ETT, and the CVP and ART-line transducers. While many surfaces in the device area had no detectable virus, multiple surfaces were positive by PCR, such as the buttons on the IV and temperature pumps (Figure 5A,B). Between all four animals the lowest Ct on any surface was 27.7, and all surfaces in the device area had high Ct values, greater than 33. 

## 4. Discussion

By providing around-the-clock care to NHPs afflicted with EVD, we were able to temporally collect and evaluate daily samples post-infection and at end-point to better understand the amount of viral RNA in such samples. Through this study we are able to better understand which procedures may or may not pose a risk to healthcare providers while managing an EVD case. 

The NHPs followed a well characterized course of disease, where viral RNA was detected between 3 and 4DPI and steadily increased until multi-organ system failure and death, at which time viral RNA was at its peak [25,26,27]. Interestingly, the amount of virus at end-point in the second animal was higher than in the first study, despite the initial infection TCID_50_ being less in the second animal (based on back titer). This suggests individual and unique courses of infection and progression of disease, as opposed end-point viremia being proportional or related to infectious dose. Ct ranges in our studies were comparable to clinical courses seen in humans [4,5,28,29]. 

Rhesus macaques as an EVD model of disease has been well described [15]. A recently published study monitored 12 Rhesus macaques for 28 days following exposure to 1000 plaque forming units (PFU) of EBOV. Death occurred by 7–10 days in all animals [30]. NHPs have been shown to have clear hallmarks of disease which mimic human disease courses including early signs such as viremia, fever, lethargy, anorexia; and late signs such as widespread systemic inflammation and disseminated intravascular coagulation, lymphocytolysis, renal tubular necrosis, and hepatocellular degeneration and necrosis. Fatal cases in humans also manifest with hypovolemic shock and multiple organ failure, as was seen in our NHP experiments [15,30]. Dose and route clearly have been shown to affect incubation time in both NHPs and humans, with the difference of several days depending on dose. However, at least in the analysis of human data from the 1976 ZEBOV outbreak, 100% of cases acquired by injection were lethal, compared to 80% of those who had a less invasive positive contact (Reviewed by [15]). In terms of relevance to the work presented here, caring for EBOV-infected patients is most risky for healthcare providers (HCPs) during late stage disease, with the exception of blood draws. Thus, as long as HCPs have appropriate PPE, clinical support, and are properly doffing contaminated PPE, risk of HCP acquisition is likely low. However, potential HCP EBOV acquisition is multifactorial, and has been thoroughly described as it pertains to clinical environments in resource-rich countries [31]. 

Our findings indicated that, as expected, blood collection is highest risk procedure, due to the viremia of the animal, and extensive care should be exercised with blood collection and handling. This includes decontamination of the syringe port following the draw, the bed area where the draw was completed, the area where manipulation occurred, and gloves of participating staff members. At present the World Health Organization recommends using two pairs of gloves while providing care and careful doffing procedures, consistent with efforts to abrogate spread via hands [32]. 

Additionally, consistent with other clinical studies, viral RNA was detected in urine at later time points compared to detection in the blood [1,33,34]; likely spillover events from endothelial cell leakage corresponding to multi-system organ failure. In our studies viral RNA was detected in the urine by 5 or 6 DPI, with exponential increase noted by 7DPI in first study, and positive post-mortem in all four studies. Bedside staff collected urine several times a day for dip and urinalysis (every eight hours), emptying the urine bag daily, flushing and Foley manipulation, which included site care and periodic re-insertion. 

We were also interested in assessing gastric residuals, related to routine tasks a provider may engage in within an ICU with an NG/nasojejunal in situ. Although clinical teams have used NG tubes during their supportive care for EVD patients [1], to our knowledge no group has assessed the viral burden in this fluid. Without the ability to check NG placement for tube feeding using a stethoscope, our team relied on checking the placement by pH monitoring (and daily *x*-rays). We also measured the gastric residual volume to assess feeding success. At times gastric residual volume could be up to 60 mL, representing a large volume of potentially infected fluid. Our findings indicate that the gastric fluid had approximately twice the viral RNA as compared to the urine at end-point, and that procedures involving this may carry exposure risk, which should be weighed against the potential benefit they may provide to the patient. Similar risks would be associated with handling/cleaning vomitus. 

We evaluated the daily clinical swabs with the intention of assessing how viral shedding influences the overall risk to the provider during the course of providing supportive care. As the highest levels were seen at the later time points of infection, when viremia was highest, we propose that the risk of mucous membrane care is most dangerous when patients are moribund, at which point such care may be less essential. Other groups have shown that while oral swabs may not be helpful to diagnose EBOV, oral swabs may be positive during disease [35], and can be used to determine infection post-mortem [36]. This is consistent with our data where only buccal swabs collected in late stage disease were positive. We collected the rectal swabs in a time-course, and the amount of viral RNA collected via the rectal swab was also not consistent between studies. Thus, assessing potential risk was related to diaper or brief changes, continuous rectal temperature, whereby the thermometer may have to be manipulated, and the insertion of suppositories (occurred within our studies). We did not detect RNA in skin swabs however this has been shown in clinical settings [35]. As we are not aware of other groups who have assessed viral RNA in skin swabs in NHPs, this may be an inherent limitation of this model where sweat is absorbed by the animal fur. 

Finally, we were surprised to find viral RNA only in certain areas in the clinical environment that did not necessarily always correlate with the highest contact surfaces. Staff removed their outer gloves following animal care and after collecting samples when possible, however at times the urgency of clinical care would prevent immediate decontamination of gloves and removal, as would occur in real-world clinical settings. The environment was also not decontaminated on a daily basis, due to restrictions in staffing resources. However, as Ebola virus peak titers are known to occur at time of death, we felt that the chances of detecting environmental positives would also peak around this time of death, as has been demonstrated elsewhere [24]. It is interesting to note that certain experiments, where there were the most intensive care interventions per unit time (e.g., including more suctions of ETT, gastric desufflations, turning of the patient from side-to side, more blood sampling—data not shown), also had higher levels of environmental contamination. Our main objective for assessing the environment at end-point was to determine the risk related to manipulating equipment as compared to patient areas, and overall device areas had less virus than patient areas. 

Although our model of EVD in NHPs demonstrates that the time from challenge to onset of viremia is only 3 days, human cases have historically shown viremia at the onset of symptoms typically between 6 and 12 days post-exposure (range 2 to 21 days [5]). This variation in human data likely takes into account the varied infectious dose and/or routes of infection, contrasting the 1000 PFU percutaneous route of challenge for this NHP model, with as low as 10 or fewer infectious particles via the mucocutaneous route suspected in some human cases. However, the clinical course, peak viral quantification, and outcome of all four animals in this experiment were remarkably similar to the final days of fatal human cases [37]. 

While our study provides insight into the risks associated with managing an EVD patient in an ICU environment, there are several limitations. First, many of the high-risk procedures completed were done before infection, including intubation and central line insertion, which have been suspected to increase transmission of high-consequence pathogens [38,39]. A second limitation is the fact that the presence of viral RNA does not necessarily indicate that infectious virus is present. However, a relationship between viral RNA quantification and infectious particles in blood has been previously demonstrated in NHPs [22,27]. We also appreciate that even if Ct is an indirect indicator of transmission risk, many of our clinical and environmental swabs have very small amounts of viral RNA, where a previous study has demonstrated increased household transmission when Cts are between 20 and 24 [40]. 

As demonstrated during the 2014 EBOV outbreak, not all healthcare providers received extensive training to provide such care and nosocomial EBOV infections have been documented both in Europe and the US [41,42]. As a result, it has been suggested that factors such as PPE shortages, lack of training, waning proficiency or improper use, inappropriate management of breaches, as well as provider fatigue are major contributors to potential nosocomial infections [31]. However, our study sheds light on the types of routine care provided that may pose the greatest risk, such as blood draws, but also demonstrates that most procedures are lower risk. This is perhaps with the exception of when the patient is in end-stage disease, and all body fluids would have detectable virus and should be handled with great care. Moreover, our data highlight the high-risk environmental areas, thereby providing information that could help healthcare workers minimize contamination of their PPE and reduce the risk of infection during removal procedures. 

## Figures and Tables

**Table 1:** Bedside care, monitoring, sample collection, and documentation *.

Task Frequency	Task Type
Hourly	Vital signs
	Ventilator settings
	Check restraints
	Verify NG placement (by measurement)
	Central line site monitoring
	Catheter site
	Pressure bags at 300
	HotDog (on and set temp)
	Cuff pressure for ventilation at 9 cc
	Accurate Ins and Outs
Every 3 h (q3h)	NG pH and volume
	Turn NHP, and shift up bed
Every 4 h (q4h)	Rinse feed bag and add feed
	Eye, nasal, and mouthcare
Every 8 h (q8h)	Accu-chek
	Urine sample and dip
	Re-zero Art and CVP lines
	Clear volumetric, syringe, and feed pumps
Daily	Change feed bag
	Record Abdominal Girth
	Chest x-ray
	Abdominal x-ray
	EKG
	CBC (HM5)
	Art Gas (ABL80)
	Venous gas (ABL80)
	MetLac 12 (Picollo)
	Biochem Plus (Picollo)
	Blood storage for viral quantification
	Gastric residual sampling and storage
	Urine sample for viral quantification
	Clinical swabs (ocular, nasal, buccal, rectal, skin)
EMR Real-Time Continuous Trending	Abdominal girth
	Vitals/Ventilator settings
	Gastric residuals
	Urine output (q4h and q24h)
	Gastric and urine pH
	Fluid balance (q24h)
	Labs (CBC, electrolytes, arterial/venous blood gases, blood glucose, albumin, liver function tests, blood urea nitrogen, creatinine).

Reproduced from [16].

## Data Availability

Not applicable.
